# Comparison of Gut Microbial Structure and Function Changes in Sichuan–Tibetan Black Pigs at Different Growth Stages Based on Metagenomic Analysis

**DOI:** 10.3390/cimb47100866

**Published:** 2025-10-21

**Authors:** Lichun Jiang, Yi Qing, Kaiyuan Huang, Huiling Huang, Chengmin Li, Qinggang Mei, Qian Wu

**Affiliations:** 1 School of Biological and Pharmaceutical Sciences, Mianyang Teachers’ College, Mianyang 621000, China; jiang_lichun@126.com (L.J.); 17760556621@163.com (K.H.); 18380527400@163.com (C.L.); scmeiqg@163.com (Q.M.); 2 School of Life Science, Mianyang Teachers’ College, Mianyang 621000, China; qyyq0924@163.com (Y.Q.); m18227952859@163.com (H.H.)

**Keywords:** intestinal microbiota, Sichuan–Tibetan black pigs, developmental stages, 16S rRNA gene sequencing

## Abstract

The gut microbiota plays a crucial role in maintaining swine health and understanding its stage-specific variations provides a scientific basis for health assessment. This study investigated the structural changes in intestinal microbiota during the development of Sichuan–Tibetan black pigs (*n* = 15) by collecting fecal samples at three growth stages: the nursery period (1 month), growing period (3 months), and finishing period (10 months). Microbial profiling was performed using 16S rRNA sequencing. Results showed no significant difference in the Shannon index between the nursery and growing periods, while the finishing period exhibited distinct ACE and Chao 1 indices compared to other stages. PCoA and NMDS analyses revealed significant structural divergence in the finishing period microbiota, with greater intra-group variability observed in the nursery and growing periods. At the phylum level, Firmicutes abundance increased progressively with growth, becoming the absolute dominant phylum, whereas Bacteroidota showed a declining trend. These characteristics are particularly prominent during the finishing period. At the family level, Lactobacillaceae abundance increased continuously. Oscillospiraceae remained stable during the early stages but decreased significantly in the finishing period. Genus-level analysis shows that Lactobacillus, especially *L. amylovorus* and *L. reuteri*, become dominant bacterial species during the finishing period. A total of 84 differentially abundant core microbiota were identified, with the finishing period containing the highest number. Functional annotation revealed 19 significantly different metabolic pathways across the three stages. The most significant is the enhanced activity of microorganisms during the finishing period in pathogen-related metabolism and exogenous degradation, reflecting their adaptability to complex feed. These findings demonstrate stage-dependent variations in the gut microbiota of Sichuan–Tibetan black pigs, providing valuable references for nutritional regulation and feeding management practices.

## 1. Introduction

The gastrointestinal tract of mammals is a complex and constantly changing ecological network, and studies have shown that gut microbiota plays an important role in physiological processes such as growth and development, nutritional digestion, and immune regulation in pigs [1]. The microorganisms in the pig gut are mainly anaerobic bacteria and facultative anaerobic bacteria, with Firmicutes and Bacteroidetes accounting for more than 90% [2]. There are significant differences in the gut microbiota of pigs at different stages. The dominant microbiota in the gut of suckling piglets are mainly lactobacilli and streptococci [3]. From lactation to weaning, the number of endogenous lactobacilli and anaerobic bacteria decreases, while the number of exogenous pathogenic bacteria such as Escherichia coli and Salmonella increases. Within 1–3 weeks after weaning [4], piglets will form a relatively stable microbial community, with bifidobacteria being the dominant microbial species in the gastrointestinal tract [5,6].

However, numerous factors can affect the composition and function of the gut microbiota, including environmental management, feed composition, use of additives, and genetic background [7]. For example, Wang et al. [8] found significant structural, compositional, and functional changes in the gut microbiota of osteoporotic rats compared to normal rats at the species level. Zhang et al. [9] found that the relative abundance of *Campylobacter* genus increased and the relative abundance of Bacteroidetes and *Streptococcus* mutans subspecies decreased in post-weaning diarrhea compared to healthy Chinese southern Anhui black pigs. Gregor et al. [10] found that the metabolome of mammals exhibits chemical diversity and is closely related to the composition of the microbiome.

At present, research on the gut microbiota of pigs is still in the exploratory stage, especially the understanding of its dynamic changes and functional succession throughout its entire lifecycle, which is far from sufficient [11]. There are differences in gut microbiota among different types of pig populations, and Sichuan–Tibetan black pigs are the most specially cultivated pig breed in Sichuan, so it is necessary to study gut microbiota at different stages [12]. Therefore, this study focuses on the structural and functional changes of gut microbiota in pigs at different growth stages, aiming to analyze the dynamic changes in gut microbiota in Sichuan–Tibetan black pigs at different developmental stages of conservation (one month), growth (three months), and fattening (ten months) through metagenomic analysis techniques, reveal their functional characteristics of gut microbiota, and provide a reference for their nutritional regulation and feeding management.

## 2. Materials and Methods

### 2.1. Sample Collection and Experimental Design

Fresh fecal samples were obtained from fifteen healthy Sichuan–Tibetan black pigs sourced from the Mianyang Jiangyou Pig Breeding Farm, with five pigs each representing the 1-month (nursery, YW), 3-month (growing, TN), and 10-month (finishing, YX) age groups and all maintained under uniform rearing conditions. The sampling involved collecting feces from each pig at three different time points within a 24 h window, which were then combined to form a single composite sample. Immediately following collection, these samples were aliquoted into sterile cryotubes, subjected to flash-freezing in liquid nitrogen, and ultimately stored at −80 °C.

### 2.2. 16S rDNA Gene Sequencing

Fecal genomic DNA was extracted from 15 pigs using a Stool Genomic DNA Extraction Kit according to the manufacturer’s instructions. The purity and concentration of the extracted DNA were assessed by agarose gel electrophoresis. The genomic DNA was then diluted to optimal concentrations for PCR amplification. During PCR amplification, the primer pair 338F (ACTCCTACGGGAGGCAGCAG) and 806R (GGACTACHVGGGTWTCTAAT) was used to target the bacterial V3-V4 hypervariable regions. The presence of PCR amplicons was confirmed by 2% agarose gel electrophoresis. Finally, the labeled fecal genomic DNA samples were shipped to Biotechnology Co., Ltd. (Jining, China) for library construction and subsequent sequencing on the Illumina MiSeq platform.

### 2.3. Bioinformatics Analysis of Sequencing Data

Raw sequencing data were preprocessed using FASTP for quality control. High-quality reads were filtered using the QIIME [13] pipeline. Valid sequences were clustered into operational taxonomic units (OTUs) at 97% similarity using the UPARSE algorithm [14], followed by taxonomic annotation against the SILVA database [15]. Alpha diversity indices (Shannon, Observed species, and ACE) were calculated using QIIME to assess microbial community richness, while beta diversity (PCoA and NMDS) was used to evaluate structural differences between groups. Sequencing depth adequacy was confirmed via rarefaction curves. LEfSe software (https://huttenhower.sph.harvard.edu/lefse, accessed on 9 October 2025) was employed to identify significantly differentially abundant taxa between groups, coupled with linear discriminant analysis (LDA) to quantify their contribution to inter-group variation [16]. Finally, to identify changes and shifts in functional gene metabolic pathways within the two groups of microbial communities, the FAAPROTAX database—widely used in microbial diversity analysis—was employed to annotate the microbial communities.

## 3. Results

### 3.1. Sample Sequencing Information

**Figure 1:**
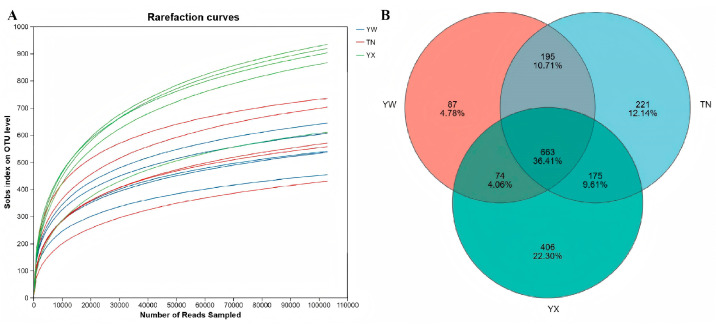
(**A**) Rarefaction curve. (**B**) Venn map of inter-group species.

To demonstrate the adequacy of the sequencing data for the samples, we conducted an analysis of the microbial alpha diversity index, Sobs, for the samples in the YW, TN, and YX groups using a rarefaction curve analysis [17]. The Sobs rarefaction curve (Figure 1A) indicates that the curve tends to flatten and eventually reaches a plateau, suggesting that the sequencing depth and coverage are sufficient for the samples to proceed to the next stage of analysis and research. A total of 15 fecal samples from the Sichuan–Tibetan black pigs at three different growth stages were collected in this study. By analyzing the sample at the OTU level with a similarity level of 97% and creating a Venn diagram, we statistically determined the number of shared and unique OTUs among the three groups. As shown (Figure 1B), there are 663 species common to all three groups, with 221, 87, and 406 unique OTUs specific to the young, nursery, and fattening stages, respectively.

### 3.2. Diversity Analysis of Gut Microbiota

**Figure 2:**
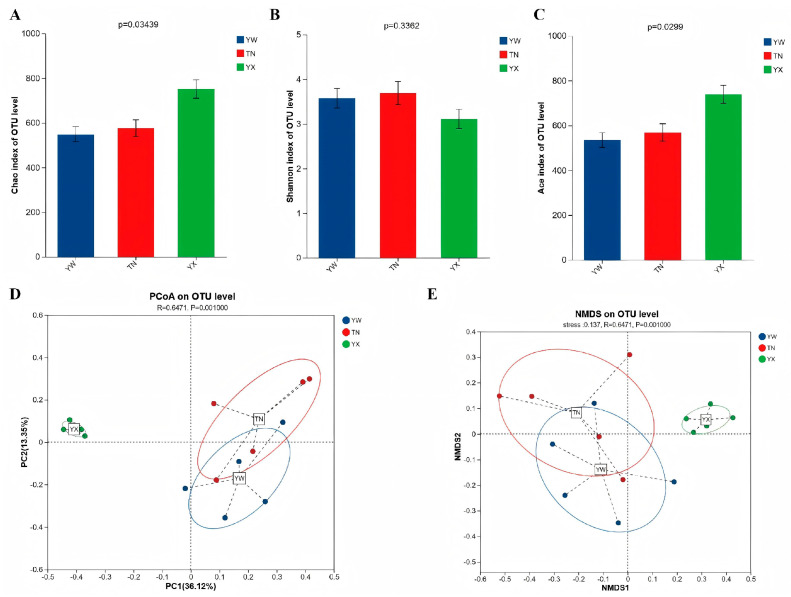
Diversity analysis of the gut microbiota. (**A**–**C**) Alpha diversity analysis of the Chao, Shannon, and Ace indices. (**D**) Principal coordinates analysis (PCoA). (**E**) Non-metric multi-dimensional scaling (NMDS).

To assess changes in the gut microbiota of Sichuan–Tibetan black pigs across three growth stages, we evaluated alpha diversity using indices of richness (Chao and Ace) and evenness (Shannon). As shown in Figure 2A–C, the YX group exhibited higher microbial evenness, reflected by an increased Shannon index, compared to the YW and TN groups. Moreover, microbial richness showed a marked increase in the YX group, as indicated by the Chao index. We further examined overall structural shifts in the microbial community using principal coordinates analysis (PCoA) and non-metric multidimensional scaling (NMDS) [18,19]. PCoA revealed clear separation among the three stages (Figure 2D), with PC1 and PC2 explaining 36.12% and 13.35% of the total variance, respectively. Similarly, NMDS confirmed substantial differences in bacterial composition and structure across the stages (Figure 2E).

### 3.3. Altered Gut Microbiota at Different Taxonomic Levels

**Figure 3:**
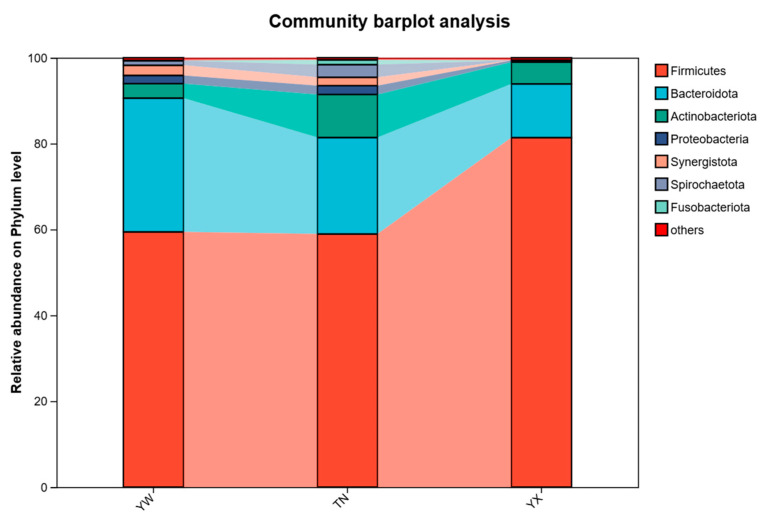
Altered gut microbiota at different taxonomic levels. Microbial composition of feces at the phylum level.

**Figure 4:**
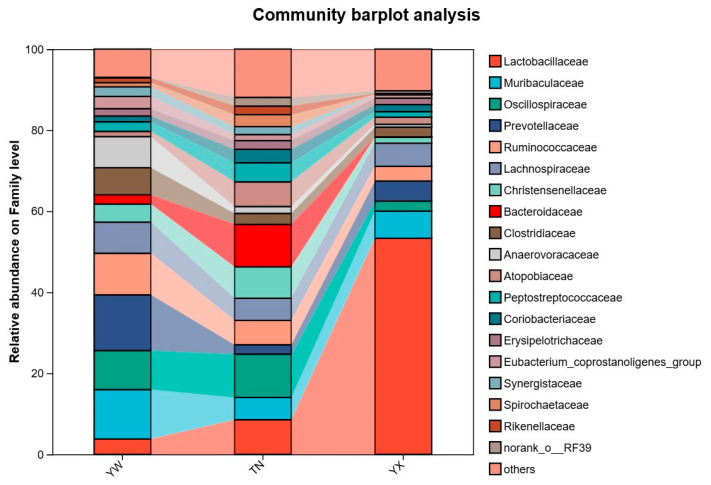
Altered gut microbiota at different taxonomic levels. Microbial composition of feces at the family level.

**Figure 5:**
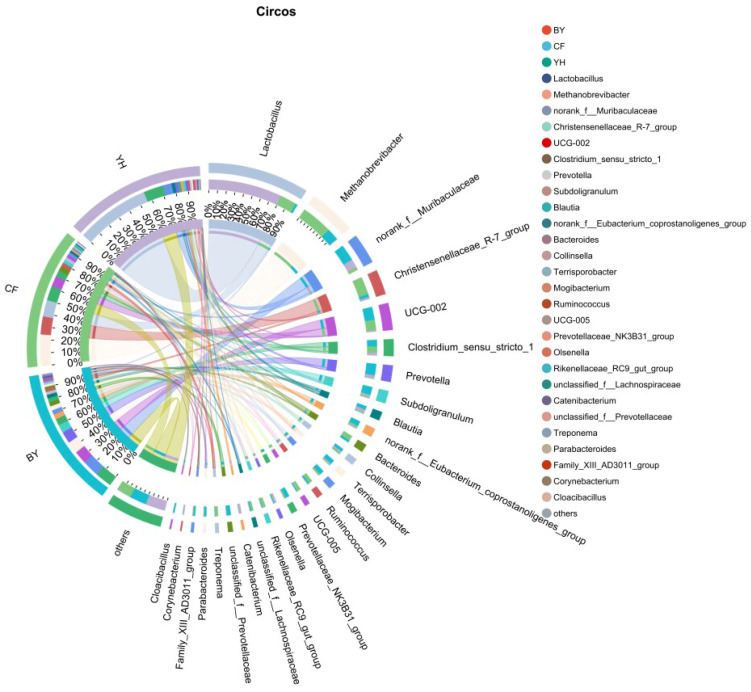
The Chord diagram displays the relative abundance of the main gut microbiota at the genes level within each group.

Consistent with the patterns observed in the PCoA and NMDS results, the relative abundances of bacteria varied across the three growth stages of the Sichuan–Tibetan black pigs at different taxonomic levels. As shown in the taxonomic classification figures (Figure 3, Figure 4 and Figure 5). At the phylum level, the dominant bacterial genera in feces at different developmental stages of pigs are Firmicutes, Bacteroidota, Actinobacteria, Proteobacteria, etc. It was found that Fusobacteria was unique to the YW and TN stages. Through the distribution of gut microbiota in pigs at different stages of phylum classification, the relative abundance of Firmicutes gradually increases with pig growth. In YX, Firmicutes dominated with a relative abundance of 81.39%, whereas its relative abundance was lower in YW and TN, at 59.43% and 58.95%, respectively. While the relative abundance of Bacteroidota shows a decreasing trend over time. The relative abundance of Actinobacteria shows an increasing trend followed by a decreasing trend over time. Through the distribution of gut microbiota in pigs at different stages at the family level, it was found that Lactobacillus were dominant, with relative abundances of 53.31% in YX, 3.77% in YW, 8.54% in TN, respectively. While the relative abundance of Muribaculaceae and Prevotellaceae showed a trend of first increasing and then decreasing over time, with relative abundances of 6.69% and 4.96% in YX, 12.19% and 13.73% in YW, and 5.49% and 2.31% in TN, respectively. Subsequently, further analysis at the genus level revealed significant differences in relative abundance in the three stages. It was found that the relative abundance of *Lactobacillus* showed a trend of first increasing and then decreasing over time, with relative abundances of 53.30% in YX, 3.77% in YW, and 8.54% in TN, respectively. Moreover, the relative abundance of *norank_f__Muribaculaceae* gradually increased with the growth of pigs, with relative abundances of 6.60% in YX, 6.01% in YW, and 5.49% in TN, respectively. The relative abundance of UCG-002 and *Christensennellacee_R-7_*group gradually decreased.

**Figure 6:**
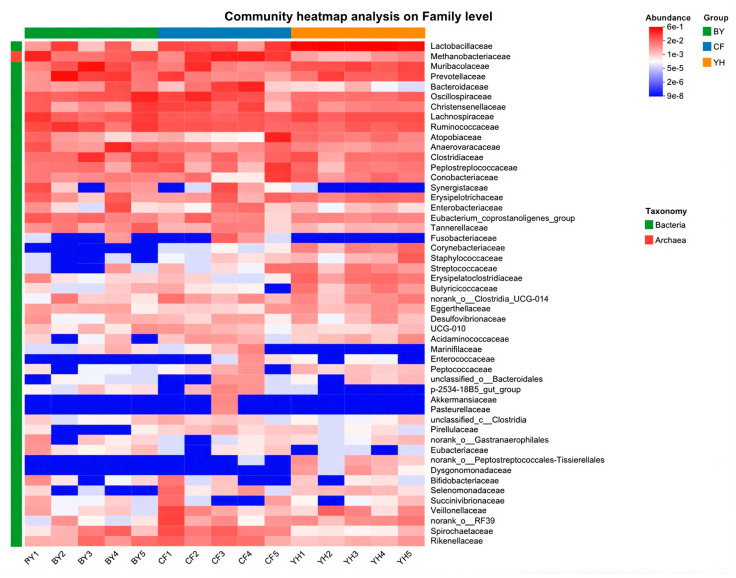
Heatmap of the top 50 most abundant gut microbial taxa at the family level.

In order to gain a deeper understanding of the impact of different growth stages on the gut microbiota of pigs, we further plotted a heatmap of the changes in the top 50 gut microbiota in terms of abundance at the taxonomic level. Among them, 18 gut microbiota showed an increase in relative abundance, while 17 gut microbiota showed a decrease in relative abundance (Figure 6). In summary, these results indicate that there are differences in the gut microbiota of Sichuan–Tibetan black pigs in the different stages.

### 3.4. Screening the Fecal Microbiota Biomarkers

**Figure 7:**
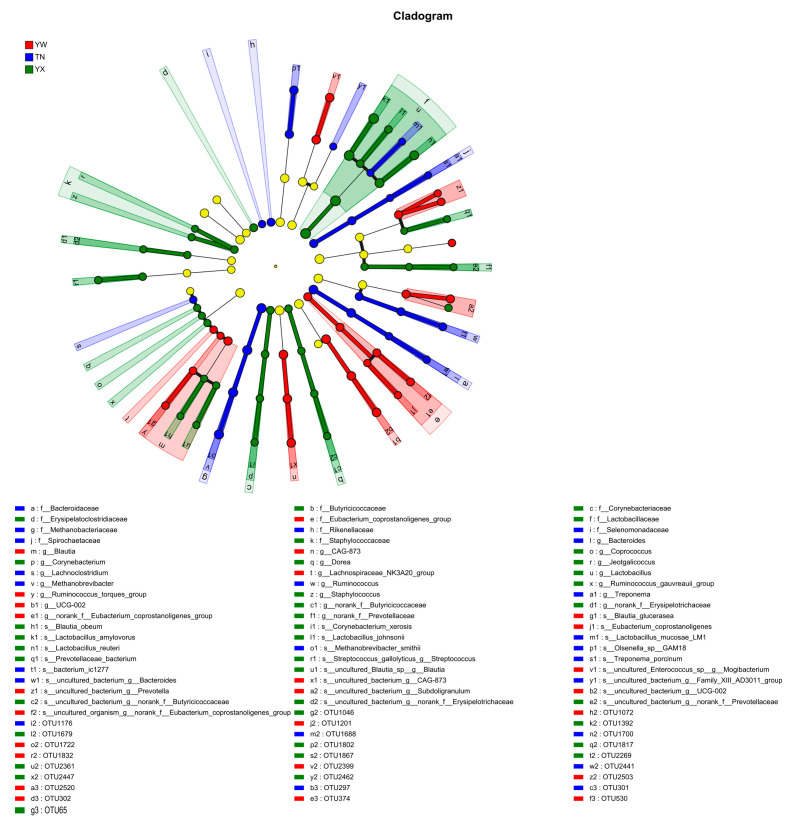
Screening the fecal microbiota biomarkers. Phylogenetic divergence analysis.

**Figure 8:**
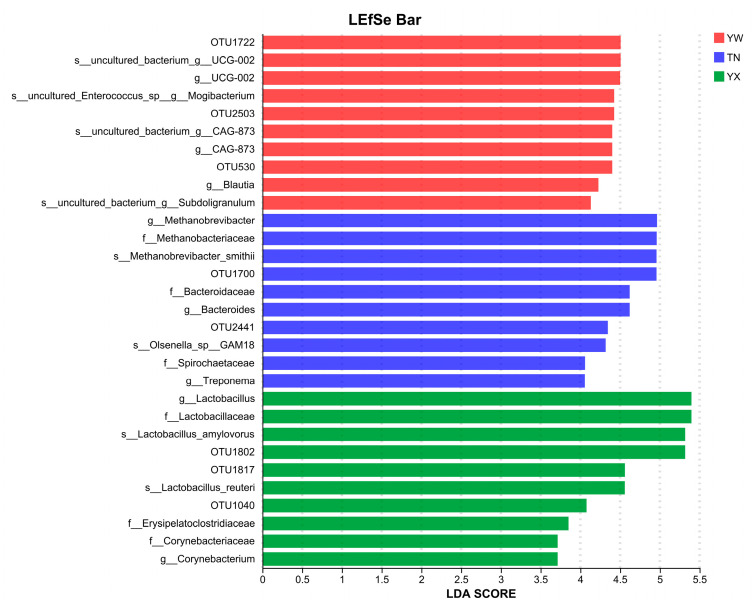
Phylogenetic divergence analysis.

An LEfSe analysis was employed to identify significantly different core microbiota in the gut of Sichuan–Tibetan black pigs across different age stages, focusing on taxa with an LDA score > 3.2. A total of 84 significantly different core microbial taxa were identified between the age groups (Figure 7). The cladogram demonstrated a significantly greater number of differentially abundant microbial taxa in the fattening stage samples compared to the nursery and growing stage samples. Based on LDA analysis (Figure 8), the top 10 microbial features with the highest LDA discriminant values were identified, and the microbial taxa with significantly different abundances in the nursery stage primarily included *g-Mogibacterium*, *g-Blautia*, and *g-Subdoligranulum*. In the nursery stage, the significantly enriched taxa were mainly *g-Methanobrevibacter*, f-Methanobacteriaceae, *s-Methanobrevibacter*, f-Bacteroidaceae, *g-Bacteroides*, *s-Olsenella*, f-Spirochaetaceae, and *g-Treponema*. In the fattening stage, the predominant differentially abundant taxa included *f-Lactobacillus*, f-Lactobacillaceae, *s-Lactobacillus amylovorus*, *s-Lactobacillus reuteri*, f-Erysipelatoclostridiaceae, f-Corynebacteriaceae, and *g-Corynebacterium*.

**Figure 9:**
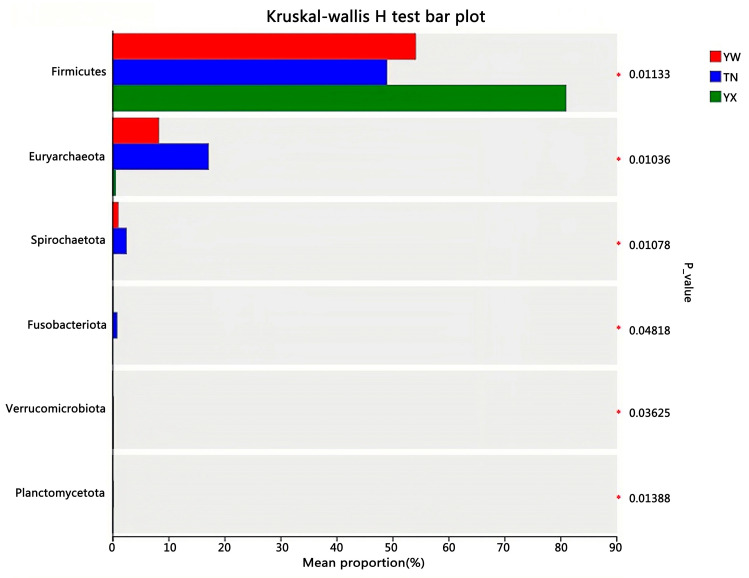
*T*-test analysis at the phylum level. * *p* < 0.05.

**Figure 10:**
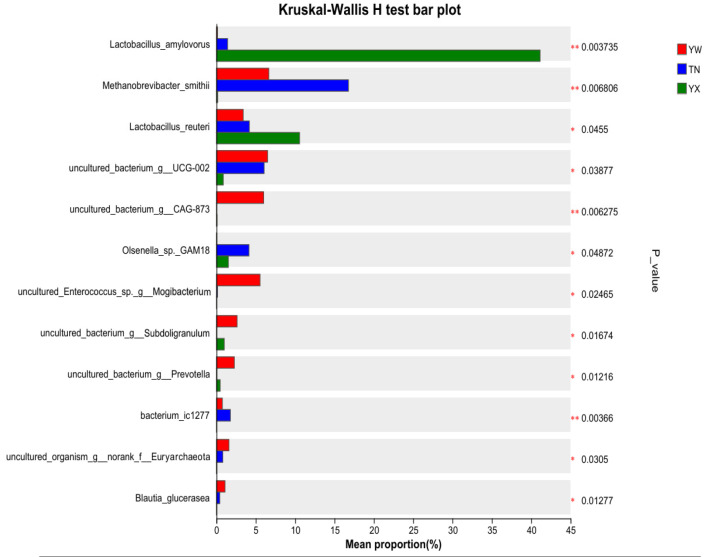
*T*-test analysis at the genus level. * *p* < 0.05, ** *p* < 0.01.

To further conduct a more comprehensive comparison of the relative abundance of candidate markers between the two groups, Wilcoxon rank-sum test analysis was primarily performed at the phylum and genus levels (Figure 9 and Figure 10). At the phylum level, the YX group demonstrated a significant elevation in Firmicutes and a pronounced decrease in Euryarchaeota relative to the other two groups. At the genus level, *Lactobacillus amylovorus* and *Lactobacillus reuteri* demonstrated a significant temporal increase, whereas *Methanobrevibacter smithii* and *Olsenella* sp. *GAM18* were significantly more abundant during the TN period than in the other two periods.

### 3.5. FAPROTAX Predictions of Gut Microbe Functions

**Figure 11:**
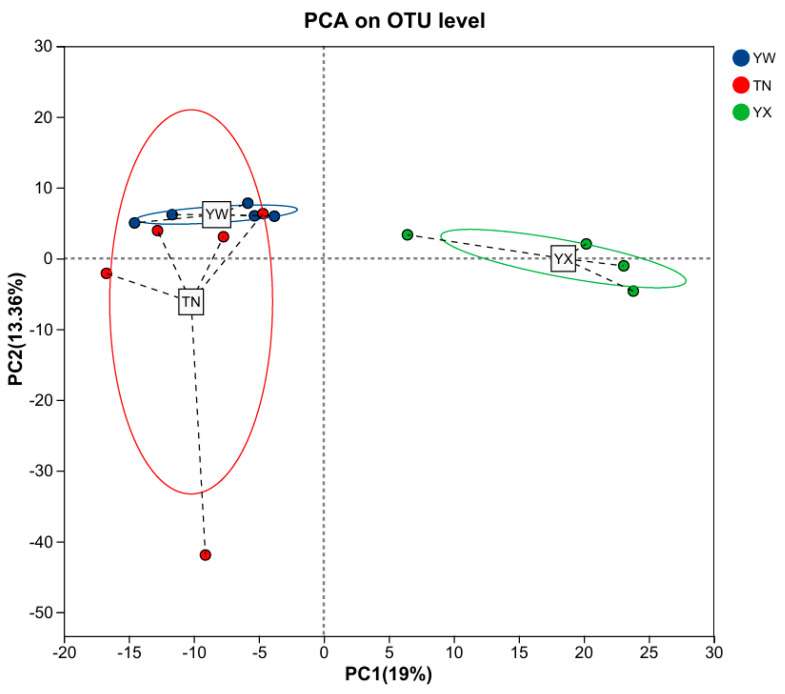
Prediction of metabolic pathways regulated by fecal microbes. Principal component analysis.

**Figure 12:**
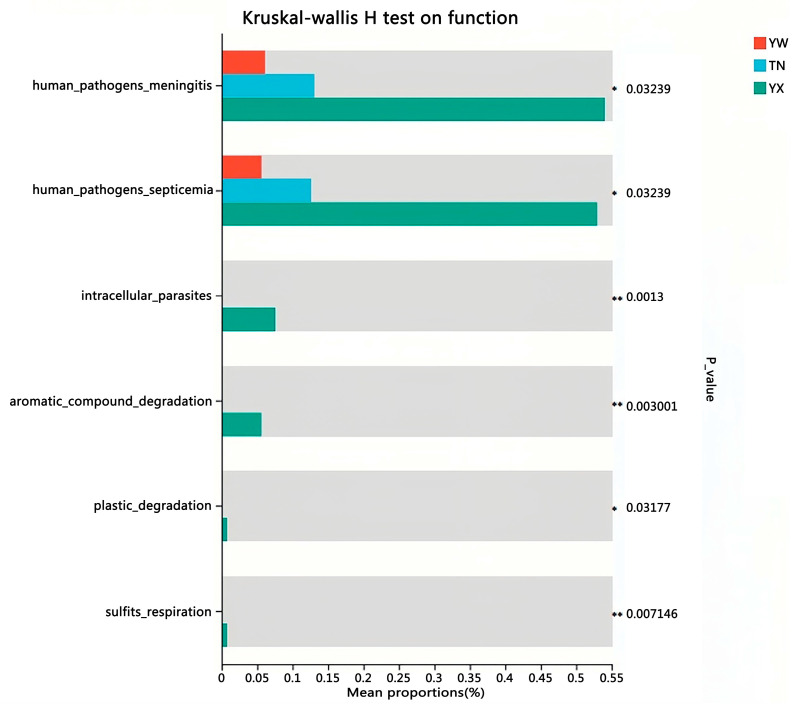
Prediction of metabolic pathways regulated by fecal microbes. FAPROTAX analysis. * *p* < 0.05, ** *p* < 0.01.

To gain more insight into the effect of different growth stages on the gut microbiota, we investigated the functional potential of the gut microbiota using FAPROTAX analysis. Predictive function richness was used to generate a principal component analysis (PCA) plot, which clearly showed a distinct clustering of samples from the YX stage compared to the other two stages, while partial overlap was observed between the YW and TN stages (Figure 11). Through implementation of FAPROTAX, we identified six metabolic pathways with the most significant differences. All pathways exhibited significant increases over time, including human pathogens meningitis, human pathogens septicemia, intracellular parasites, aromatic compound degradation, plastic degradation, and sulfite respiration (Figure 12).

## 4. Discussion

The gut microbiota is a complex and intricate ecosystem composed of bacteria, fungi, and viruses, with a wide variety and large quantity. Its composition is not fixed but is influenced by various factors including diet, age, stress, and environment, which is of great significance for the acquisition and composition of pig gut microbiota [20]. This study explored the temporal patterns of changes in pig microbiota and revealed the spatiotemporal dynamic distribution of pig gut microbiota.

Our results are also supported by previous findings. Alpha diversity analysis revealed comparable richness between the nursery (YW) and growing (TN) periods, while the finishing period (YX) exhibited increased richness but slightly reduced diversity (non-significant). This is contrary to previous reports suggesting progressive microbial diversification with age [21,22]. Our conclusions are highly similar to those of another study focusing on local black pigs. He et al. [23] found that the fecal microbial diversity and composition of Chuanxiang black pigs exhibit dynamic changes during their growth and development. Some specific bacterial strains that appear at certain developmental stages of Chuanxiang black pigs, such as Prevotella copri, may have influenced the formation of their excellent traits. This confirms the rationality of our conclusions. These findings also support Yang et al. [24] who showed the relationship between pig breeds and gut microbiota structure and found that the gut microbiota composition of different breeds of pigs varies to some extent. In the detection of pig manure samples and digestive tract contents, the number of total bacteria, Firmicutes, Bacteroidetes, and sulfur-reducing bacteria in adult pig manure samples of different breeds varies.

Beta diversity and compositional analyses demonstrated marked structural shifts between TN and YX phases, which is likely attributable to weaning stress. Piglets typically undergo weaning at 20 days, with the YW and TN periods representing critical post-weaning adaptation phases. The transition from highly digestible sow milk to coarse, less palatable solid feed [25] drives gastrointestinal remodeling and microbial community instability. These findings also support the findings of Zhu et al. [26] who found through 16S rRNA sequencing of the gut microbiota of piglets before and after weaning that weaning has a significant impact on the gut microbiota of piglets. After weaning, the gut microbiota of piglets changed after consuming the newly introduced diet.

Our research also found that at the phylum level, Firmicutes, Bacteroidota, Actinobacteria, and Proteobacteria dominated all stages, aligning with prior porcine microbiota studies [27,28]. For example, Tap et al. [29] found that in the gastrointestinal tract of monogastric animals such as pigs, Proteobacteria, Firmicutes, and Bacteroidetes are the dominant bacterial groups at the phylum level. In animals, fibroblast-derived succinic acid bacteria, white rumen bacteria, yellow rumen bacteria, fiber-bending bacteria, and Prevotella are dominant bacterial groups at the genus level in the rumen [17]. However, subtle variations suggest breed-specific microbial stratification. Notably, genus-level analysis revealed dramatic expansion of Lactobacillus amylovorus and Lactobacillus reuteri in YX. This correlates with the metabolic shift from milk oligosaccharide to plant polysaccharide degradation post-weaning [30]. Lactobacillus spp. [31] enhance energy harvest via lactic acid fermentation from carbohydrates, while the U-shaped trajectory of Prevotellaceae abundance mirrors dietary fiber adaptation; Prevotella-dominant microbiota associates with long-term high-fiber intake [32], which is consistent with post-weaning solid feed utilization.

Functional annotation of the pig gut microbiota revealed 19 significantly differential metabolic pathways among the YW, TN, and YX stages, 12 of which peaked during the finishing period as shown in the bar chart. There are related studies that are very similar to the results of this study. Li et al. [33] found that Tibetan pigs have rich and unique microbial composition and functional pathways. The phylum Verrucomicrons and Akmansia, which are related to immunity and disease resistance, are high-abundance bacterial genera unique to the intestinal tract of Tibetan pigs, which may be related to their adaptation to high-altitude, low-oxygen, and low-temperature environments and their disease resistance. The LEfSe results of Himansu et al. [34] also support our findings, as some metabolic pathways, such as energy metabolism, differ in two different stages of the cecal microbiota. Multivariate analysis conducted through PCA and Sparse Partial Least Discriminant Analysis (sPLS) in two stages also showed similar metabolic pathways, such as amino acid metabolism, human diseases, and genetic information dominating in both stages.

## 5. Conclusions

This study employed 16S rRNA sequencing of fecal samples from the nursing, growing, and fattening stages to elucidate dynamic changes in gut microbial functional structure. Alpha and beta diversity analyses revealed that microbial composition was similar with marked inter-individual variations between the nursing and growing stages, whereas the fattening stage exhibited distinct richness and compositional divergence. At the phylum level, Firmicutes, Bacteroidota, Actinobacteriota, and Proteobacteria consistently dominated across all stages. Conversely, the family and genus levels demonstrated significant stage-dependent shifts in dominant strains. A total of 84 differentially abundant core microbial taxa were identified among developmental stages, with the highest divergence observed in the fattening stage—a finding in line with its altered microbial richness.

This study not only elucidates the developmental patterns of pig gut microbiota, providing a microbiological basis for healthy farming and disease prevention and control, but also establishes an experimental method system that can provide technical references for the study of animal microbiota and has guiding value for optimizing feeding management and promoting high-quality development of the pig industry.

## Data Availability

The original contributions presented in this study are included in the article. Further inquiries can be directed to the corresponding author.
